# Effect of Different Female Factors on Pregnancy Rates in Artificial Insemination With Donor Sperm Cycles: A Prospective Observational Study

**DOI:** 10.7759/cureus.95989

**Published:** 2025-11-03

**Authors:** Shubhangi Juneja, Padmalaya Thakur, Jyotiranjan Sahoo, Basanta Kumar Pati, Sujata Pradhan

**Affiliations:** 1 Department of Obstetrics and Gynaecology, Institute of Medical Sciences and SUM Hospital, Siksha ‘O’ Anusandhan, Deemed to be University, Bhubaneswar, IND; 2 Center for Human Reproduction, Institute of Medical Sciences and SUM Hospital, Siksha ‘O’ Anusandhan, Deemed to be University, Bhubaneswar, IND; 3 Department of Community Medicine, Institute of Medical Sciences and SUM Hospital, Siksha ‘O’ Anusandhan, Deemed to be University, Bhubaneswar, IND

**Keywords:** aid, artificial insemination, donor sperm, female factors, intrauterine insemination

## Abstract

Background

Artificial insemination with donor sperm (AID) is an established treatment option for various indications. The outcome of such treatment is likely to be affected by different female factors and associated factors contributing to infertility. Existing evidence lacks clarity in this regard. This study aimed to assess the effect of different female factors on pregnancy rates in donor insemination cycles. This prospective observational study was conducted in a tertiary care teaching hospital from March 2023 to December 2024.

Methods

One hundred and two patients undergoing AID cycles were included in the study. Various female characteristics were compared for pregnancy outcomes. Data entered in Microsoft Excel (Microsoft® Corp., Redmond, WA) and analysed using Statistical Product and Service Solutions (SPSS, version 27.0; IBM SPSS Statistics for Windows, Armonk, NY). Statistical association was tested using the chi-square test. A p-value < 0.05 was statistically significant.

Results

Out of 102 females undergoing AID cycles, 15 had a clinical pregnancy (14.7%). Ongoing pregnancy rate was 11.8%. The duration of infertility was significantly less in patients having successful clinical and ongoing pregnancies (p<0.05). Female age, body mass index (BMI), number of AID cycles, type of infertility, and additional female factors affecting fertility were similar in patients with or without successful pregnancies.

Conclusion

Clinical and ongoing pregnancy rates after AID cycles are affected by the duration of infertility. The presence of polycystic ovary syndrome (PCOS) and endometriosis in female partners is less likely to alter the AID cycle outcomes.

## Introduction

Artificial insemination with donor sperm (AID) is considered an easy, affordable, and widely available treatment for infertility due to specific indications. Couples with severe male factor infertility are the primary beneficiaries, as male factor contributes to infertility in 50% of couples, alone or in combination with female factors [1]. In vitro fertilization (IVF) is the standard fertility treatment for male infertility. When IVF treatment is not feasible due to financial reasons or failure of sperm retrieval procedures, couples are advised to undergo intrauterine insemination (IUI) with donor sperm. This treatment is also indicated in case of male partners with Y chromosome-linked diseases, as it carries the risk of transmission to the male offspring [2]. Moreover, the Assisted Reproductive Technique (ART) Regulation Act 2021 recognises single mothers to be eligible for ART [3]. Hence, the outcome of AID treatment needs optimisation and fine-tuning, as for many couples it remains an important treatment option.

Researchers vary in their opinions regarding factors affecting the outcome of AID. The total motile fraction (TMF) of spermatozoa is an important parameter to predict AID outcome [4]. While factors such as female age have been extensively studied in this context, some authors consider it to be an effective predictor [5-7]. However, many studies fail to demonstrate the effect of female age on AID success [8,9]. Other than age, the effect of other female factors on the AID outcome lacks detailed analysis. Since the male partner is compromised in most couples choosing AID, the contribution of female parameters needs more attention, as she is the sole partner participating in the treatment. Moreover, the sperm donors are selectively chosen and assumed to be healthy males. Endometriosis and polycystic ovary syndrome are two major health concerns in reproductive-age females. The prevalence of polycystic ovary syndrome (PCOS) varies from 4% to 21% depending on the diagnostic criteria used [10]. Similarly, approximately 10% of females in the reproductive age group have endometriosis [11]. Hence, the presence of these factors in females undergoing AID is not uncommon and warrants evaluation. While many large studies contributing to evidence are retrospective in nature, the present prospective study aims to demonstrate the effect of various female parameters that may influence the pregnancy rate after AID [12,13]. The study specifically emphasizes including every participant for a single cycle only at any point during their treatment so that each observation will be independent to get a valid result.

## Materials and methods

This prospective observational study was conducted in a tertiary care teaching hospital from March 2023 to December 2024. Institutional ethics committee approved the study (Ref No./IEC/IMS.SH/SOA/2022/955). Guidelines for the protection of human subjects were strictly adhered to as per the Helsinki Declaration 2013. Informed written consent was obtained from all the study participants before enrolling them in the study. According to the study by Thijssen et al., the pregnancy rate was 53.73% with a 95% confidence interval of 48.72%-58.68% [7]. Considering this effect size with an alpha of 5% and a power of 80%, the sample size was calculated to be 96. The sample size was calculated using OpenEpi software version 3.

Couples scheduled for artificial insemination with donor sperm were recruited for the study on the day of IUI. Each patient was enrolled in the study once only, irrespective of the number of AID cycles. Patients receiving ovarian stimulation with letrozole and human menopausal gonadotropin (hMG) and human chorionic gonadotropin (hCG) as an ovulation trigger before IUI were considered for inclusion in the study. Females ≥40 years, BMI ≥35 kg/m^2^, and patients receiving different ovarian stimulation protocols or gonadotropin agonists as ovulation trigger were excluded. Before the procedure, all subjects underwent a complete clinical evaluation with detailed menstrual, obstetric, sexual, and personal history. Demographic and clinical parameters such as age, type and duration of infertility, and number of artificial insemination cycles were noted. Height, weight, and body mass index (BMI) of female partners were measured. PCOS was diagnosed by an ultrasound performed during evaluation as per the Rotterdam criteria [14]. Diagnosis and staging of endometriosis were done during laparoscopy as per revised American Fertility Society criteria [15]. The patients with at least one patent fallopian tube documented in any of the standard diagnostic modalities were eligible for AID. The objective of the study was to analyse the effect of different female characteristics and additional infertility factors on the pregnancy rate after AID treatment. Ongoing pregnancy rate was considered the primary outcome, and clinical pregnancy rate was the secondary outcome for the present study.

Controlled ovarian stimulation was performed with letrozole (Letroz, Sun Pharma Laboratories, India) 2.5 or 5 mg daily from the second or third day of the menstrual cycle for five days. Further ovarian stimulation was done by adding injection hMG (75 IU; GMH, Sun Pharma Laboratories, India) on cycle days five, seven, and nine. The dose of letrozole was determined after considering various factors such a age, BMI, ovarian reserve, and follicular response in previous cycles. Follicular monitoring was done by transvaginal ultrasonography on cycle day 10 or 11. Following the detection of one or two dominant follicles greater than 18 mm in size, HCG 10,000 IU was administered intramuscularly to trigger ovulation. Insemination was done 36-40 hours after the ovulation trigger. The cycle was cancelled in the presence of more than three follicles greater than 15 mm in size to avoid ovarian hyperstimulation syndrome and multiple gestation.

A pre-processed frozen donor sperm sample procured from a registered ART bank was used for the treatment. It was thawed initially at room temperature (25°C) for 30 minutes and then warmed at 37°C for 15 minutes. Post-thaw motility was checked, and the TMF was calculated. Approximately 0.5 mL of processed sperm sample was loaded in a soft IUI catheter (Manish Medi Innovation, India). The insemination process was performed with the patient in the lithotomy position. The cervix was exposed with Cusco's speculum, and the thawed donor sperm sample was injected gently into the uterine cavity loaded in the IUI catheter. The patient was advised to lie down for 15 minutes after insemination.

Micronized progesterone 200 mg (Susten, Sun Pharma Laboratories, India) vaginal pessaries were advised for twice daily use for 15 days post-procedure as luteal phase support. A urine pregnancy test (UPT) was done 20 days after insemination to diagnose biochemical pregnancy. Transvaginal ultrasonography was done two weeks after a positive UPT to confirm a clinical pregnancy by identifying the gestational sac and/or embryonic pole. Each pregnancy was followed at least till 12 weeks to confirm an ongoing pregnancy.

Statistical analysis

Data entered in Microsoft Excel (Microsoft® Corp., Redmond, WA) and analysed using Statistical Product and Service Solutions (SPSS, version 27.0; IBM SPSS Statistics for Windows, Armonk, NY). The categorical variables were expressed as frequency and percentage, while continuous variables were expressed as median and interquartile range (IQR). The normality of the variables was assessed using the Shapiro-Wilk test. The association between two categorical variables was determined using Fisher’s exact test. Comparison between two medians was assessed using the Mann-Whitney U test. A p-value of < 0.05 was considered statistically significant.

## Results

Data were analyzed for 102 AID cycles performed during the study period. Baseline characteristics of the study population are summarised and presented (Table 1). Age, duration of infertility, and number of AID cycles were non-normally distributed, hence expressed as median and IQR. The median age of patients was 29 (25.0-34.0) years, the median duration of infertility was 4.0 (2.0-6.0) years, and the median AID cycle number was 1.57 (1.0-2.0). The mean BMI was 25.08±3.21 kg/m^2^. The categorical variables were represented as frequency and percentage. Eighty-four (82.4%) cases had primary infertility, and 18 (17.6%) had secondary infertility. Eleven (10/8%) females had PCOS, while endometriosis was diagnosed in six (5.9%) patients. In the remaining 85 (83.3%) patients, no obvious female infertility factor was noted. The outcome of the study in terms of pregnancy rate is represented in Table 2. Twenty (19.6%) patients had biochemical pregnancies. Fifteen (14.7%) had ultrasonographically confirmed pregnancies at six to seven weeks. Twelve patients (11.8%) were observed to continue their pregnancies beyond 12 weeks.

**Table 1:** Baseline characteristics of the study population PCOS-Polycystic Ovary Syndrome, AID-Artificial insemination with donor sperm, BMI- Body Mass Index, IQR-Interquartile Range SD-Standard Deviation

Variables	Mean/Median/Frequency	SD/IQR/percentages
Non-Normally distributed variables (Median and IQR)		
Age in years	29	25.0 - 34.0
Duration of infertility in years	4	2.0 – 6.0
AID cycle number	1.57	1.0 – 2.0
Normally distributed variable (Mean and SD)		
BMI in Kg/m^2^	25.08	3.21
Categorical variables (Frequency and Percentage)		
Type of infertility		
Primary	84	82.4
Secondary	18	17.6
Associated female factor		
PCOS	11	10.8
Endometriosis	6	5.9
None	85	83.3

**Table 2:** AID cycle outcome in terms of pregnancies AID-Artificial Insemination With Donor Sperm

Outcome	Frequency	Percentage (%)
Biochemical pregnancies	20	19.6
Clinical pregnancies	15	14.7
Ongoing pregnancies	12	11.8
Miscarriages	Mar-15	20

Pregnancy outcomes were assessed in terms of biochemical, clinical, and ongoing pregnancy rates. Twenty (19.6%) biochemical pregnancies were documented. Clinical pregnancy was confirmed for 15 (14.7%) women in ultrasonography, and ongoing pregnancy was documented in 12 (11.8%) cases. Three (20%) patients had miscarriages before 12 weeks. Baseline characteristics were compared between the patients with and without clinical pregnancies (Table 3). The mean age was similar for both categories of patients (27.6±4.17 vs 30.13±4.98; p=0.063). Similarly, they had similar BMI (24.49±2.31 vs 25.8 ±3.34; p=0.44). The patients with successful clinical pregnancies had a short duration of infertility. The AID cycle number did not show statistically significant associations with the clinical pregnancy rate. Thirteen patients out of 15 (86.7%) with clinical pregnancy had primary infertility, and two (13.3%) had secondary infertility. A similar trend was also observed for the patients who did not have confirmed pregnancies (p=0.999). There was no association observed between female infertility factors and clinical pregnancy rate (p=0.616). Thirteen out of 15 patients with successful AID cycles did not have any associated female factor (86.7%), two (13.3%) had PCOS, and none had endometriosis. Similarly, 72 out of 87 (82.8%) patients without clinical pregnancies did not have an associated female factor, nine (10.3%) had PCOS, and six (6.9%) had endometriosis. This observation is not statistically significant.

**Table 3:** Association of different factors with clinical pregnancy #Values show mean±SD, ##Value expressed in median and inters quartile range, and ###Values show frequency and percentage. Statistical significance was determined at p < 0.05. p-values < 0.05 are denoted with an asterisk (*).

Variables	Yes	No	Test value	P value
Age in years#	27.6±4.17	30.13±4.98	849	0.063
BMI in kg/m^2^#	24.49±2.31	25.8±3.34	4.98	0.443
Duration of infertility in years#	3.16±1.06	4.79±2.83	862.5	*0.044
AID cycle number##	1.0 (1.0-2.0)	1.0 (1.0-2.0)	695	0.633
Type of infertility###	13 (86.7%)	71 (81.6%)	0.225	0.999
Primary				
Secondary	2 (13.3%)	16 (18.4%)		
Female factor###	2 (13.3%)	9 (10.3%)	0.769	0.616
PCOS				
Endometriosis	0 (0%)	6 (6.9%)		
None	13 (86.7%)	72 (82.8%)		

The association of ongoing pregnancy with different variables was analysed and represented in Table 4. Like clinical pregnancy, the ongoing pregnancy rate had a significant association with the duration of infertility. Patients with ongoing pregnancies had a shorter duration of infertility than the rest of the patients (2.87±0.85 vs 4.7±2.7; p=0.015). There was no association of ongoing pregnancy with female age, BMI, or AID cycle number. Associated female infertility factors such as endometriosis and PCOS also did not show a significant association with ongoing pregnancy (p=0.673). None of the patients with successful ongoing pregnancies had endometriosis, whereas six out of 90 (6.7%) patients without ongoing pregnancy had endometriosis. Two out of 12 (16.7%) patients with ongoing pregnancies were known cases of PCOS, and nine out of 90 (10.0%) females without ongoing pregnancy had PCOS. Ten out of 12 (83.3%) of patients with ongoing pregnancies had no additional female factors contributing to infertility diagnosed before the procedure. A similar proportion of patients (75/90, 83.3%) without successful ongoing pregnancies did not have any female infertility factors.

**Table 4:** Association of different factors with ongoing pregnancy #Values show mean±SD, ##Value expressed in median and inters quartile range, and ###Values show frequency and percentage. Statistical significance was determined at p < 0.05. p-values < 0.05 are denoted with an asterisk (*).

Variables	Yes	No	Test value	P value
Age in years#	27.25±4.20	30.09±4.95	726.5	0.052
BMI in kg/m^2^#	24.56±2.54	25.15±3.30	2.506	0.555
Duration of infertility in years#	2.87±0.85	4.7±2.7	771.5	*0.015
AID cycle number##	1.0 (1.0-2.0)	1.0 (1.0-2.0)	530	0.902
Type of infertility###	10 (83.3%)	74 (82.2%)	0.009	0.999
Primary				
Secondary	2 (16.7%)	16 (17.8%)		
Female factor###	2 (16.7%)	9 (10.0%)	0.968	0.673
PCOS				
Endometriosis	0 (0)	6 (6.7%)		
None	10 (83.3%)	75 (83.3%)		

## Discussion

This study was conducted to demonstrate the effect of common female parameters and infertility factors on the AID success. Clinical pregnancy rate was 14.7%, which lies within the range of 13%-22.3% documented by other authors [6,7,16]. Ongoing pregnancy rate was 11.8%. As the study was part of a postgraduate dissertation, it was not possible to follow up the pregnancies for live birth due to the restricted time frame for research. Among the female parameters, the age of the female did not affect the clinical and ongoing pregnancy rate. It was negatively associated with pregnancy rate after AID in two other studies [17,18]. The differences in observation may be due to the small sample size in the current study. We excluded AID cycles performed at or after 40 years of age, as the chances of pregnancy from AID were rare beyond 40 years [19]. In the later study, the maximum included female age was 45.9 years. In the study by Bahadur et al., the pregnancy rate after an AID cycle was also not affected by female age [8]. Many authors have found a negative impact of BMI on homologous IUI cycles [20,21]. The difference in observation for BMI in this study may be due to a smaller number of obese patients undergoing AID treatment and exclusion of females with BMI ≥ 35 kg/m^2^. A systematic review and meta-analysis of 11 studies also demonstrated no effect of BMI on pregnancy rate after IUI [22].

The study observed a negative association of duration of infertility with clinical and ongoing pregnancy rates. A similar effect was also demonstrated in another study, in which the pregnancy rate was lower if the duration of infertility was more than four years [23]. In the present study, the mean duration of infertility with failed AID cycles was 4.79 years. The number of AID cycles was similar irrespective of the cycle outcome. A similar observation was noted in the study by Kang et al. till the seventh cycle of AID [5]. The present study included AID cycles up to the sixth attempt.

Since PCOS and endometriosis are known causes of female infertility, they are likely to affect the cycle outcomes after AID. In this study, only minimal and mild endometriosis were considered for IUI. Females with moderate and severe endometriosis were encouraged to undergo IVF after diagnosis by laparoscopy. In a study of 480 homologous IUI cycles, the pregnancy rate was similar in patients with anovulation and endometriosis [24]. Minimal and mild endometriosis did not affect IUI success compared to unexplained infertility in another study [25]. In that study, the pregnancy rate was grossly reduced in severe endometriosis. In a study by Aly et al., no difference in pregnancy rate was observed in patients with PCOS undergoing IUI cycles compared to the timed intercourse group [26].

The current study is the first of its kind to analyse the effect of female infertility factors such as PCOS and endometriosis on pregnancy rate in AID cycles. The lack of difference in pregnancy rate was explained by the fact that the study included only minimal and mild endometriosis cases, like other studies on IUI cycles. The current study has the advantage of being a prospective study. There was no loss to follow up patients, as the enrolment was done on the day of insemination. A uniform ovarian stimulation protocol was followed to avoid the difference in pregnancy rate due to different ovarian stimulation protocols. In the present study, the per-patient pregnancy rate was determined, as we had recruited one patient only once, irrespective of the number of AID cycles. This was done to make each observation independent to make a valid inference. At the same time, the study declares some drawbacks. The sample size was small compared to most of the retrospective studies in the literature. Patients were followed up only for clinical and ongoing pregnancies, whereas live birth rate could not be assessed due to the limited time frame of the study.

## Conclusions

As per the observations of the current study, a long duration of infertility negatively affects the pregnancy rate after AID. In females less than 40 years with a BMI below 35 kg/m^2^, the number of IUI cycles and the type of infertility do not impact the success rate of this procedure. Additional female infertility factors, such as PCOS or minimal and mild endometriosis, are unlikely to affect the outcome of AID. Though this is a prospective study, another study with a large sample size may be able to demonstrate the impact with more accuracy. Future studies evaluating the effect of these parameters on live birth rate after AID are also warranted.
